# The impact of inclusive mentoring and identity work on self-efficacy in career advancement and career commitment among underrepresented early-career faculty and post-doctoral fellows

**DOI:** 10.1017/cts.2024.504

**Published:** 2024-03-22

**Authors:** Maya S. Thakar, Doris M. Rubio, Audrey J. Murrell, Natalia E. Morone, Chantele Mitchell Miland, Gretchen E. White

**Affiliations:** 1 Institute for Clinical Research Education, University of Pittsburgh Schools of the Health Sciences, Pittsburgh, PA, USA; 2 College of Business Administration, University of Pittsburgh, Pittsburgh, PA, USA; 3 Department of Medicine, Boston University Chobanian & Avedisian School of Medicine, Boston, MA, USA; 4 Boston Medical Center, Boston, MA, USA

**Keywords:** Science identity, researchers who are underrepresented in biomedical sciences, career progression, diversifying the biomedical research workforce, career commitment, self-efficacy in career advancement

## Abstract

**Objective:**

Researchers from underrepresented groups leave research positions at a disproportionate rate. We aim to identify factors associated with self-efficacy in career advancement and career commitment among underrepresented post-doctoral fellows and early-career faculty.

**Methods:**

Building Up is a cluster-randomized trial with 25 academic health institutions. In September-October 2020, 219 Building Up participants completed the pre-intervention assessment, which included questions on demographics, science identity, mentoring, self-efficacy in career advancement (i.e., advancement is open to me, confidence in career progression, confidence in overcoming professional barriers), and career commitment (i.e., intent to continue research training or studying in a field related to biomedical sciences). Using logistic and multinomial logistic regression, we identified characteristics independently associated with self-efficacy in career advancement and career commitment.

**Results:**

The cohort is 80% female, 33% non-Hispanic/Latinx Black, and 34% Hispanic/Latinx. Having mentors that address diversity was significantly associated with the belief that advancement is open to them (OR = 1.7). Higher science identity (OR = 4.0) and having mentors that foster independence (OR = 1.8) were significantly associated with confidence in career progression. Higher science identity was also significantly associated with confidence in overcoming professional barriers (OR = 2.3) and intent to continue studying in a field related to biomedical sciences (OR = 3.3). Higher age (OR = 2.3) and higher science identity (OR = 4.2) were significantly associated with intent to continue research training.

**Discussion:**

Science identity and mentoring play key roles in self-efficacy in career advancement and career commitment. These factors may contribute to retention of underrepresented early-career biomedical researchers.

The lack of racial and ethnic diversity in the biomedical research workforce and the disproportionate rate at which researchers from underrepresented groups in the biomedical sciences leave research positions are well-documented [1,2]. Researchers from groups underrepresented in academic medicine encounter more obstacles (i.e., high demand of clinical duties, promotional disparities, and social isolation) in their work environments compared to their well-represented counterparts [3,4], and regularly face racism and discrimination in the workplace [3].

Faculty from underrepresented groups are also slower to progress in their career [5]. For example, underrepresented faculty midwives and nurses work in early-career-level positions (i.e., assistant professor) for approximately 6 years, almost three years longer than White faculty midwives and nurses [5]. Existing literature emphasizes the need for interventions tailored toward employees from groups underrepresented in science-related fields to improve career progression [5]; however, factors associated with career advancement among researchers from underrepresented groups are unclear. It is important to identify factors associated with career commitment and self-efficacy in career advancement among groups underrepresented in biomedical research to develop effective methods to increase retention of these researchers. Prior research shows that mentoring and engaging in positive identity work are key to supporting positive career outcomes for underrepresented groups [6]. Therefore, we aimed to identify factors associated with self-efficacy in career advancement and career commitment among post-doctoral fellows and early-career faculty who are from groups underrepresented in biomedical sciences.

## Methods

### Design and participants

This manuscript describes pre-intervention data (collected via REDCap in September and October 2020) from both intervention arms of the Building Up trial. Building Up was a cluster-randomized trial that took place at 25 academic institutions (Supplemental Figure 1) throughout the United States. It aimed to evaluate the effectiveness of an intervention on research success of 224 post-doctoral fellows and early-career faculty from groups underrepresented in the biomedical sciences [7,8]. According to the National Institutes of Health, people who are underrepresented in science include individuals from racial or ethnic groups identified as underrepresented in biomedical sciences, individuals with disabilities, and individuals from disadvantaged backgrounds [9,10]. The trial had two intervention arms that lasted 10 months; each intervention arm consisted of four components: monthly sessions, mentoring, networking, and coursework [11]. All participants were given the opportunity to attend monthly leadership webinars [11]. Participants in the “high touch” intervention arm participated in monthly meetings with study-assigned near-peer mentors and fellow participants to discuss the hidden curriculum in academia; experienced intervention-provided near-peer mentoring; participated in networking opportunities through an orientation and poster sessions; and completed coursework in grant and scientific writing [11]. Participants in the “low touch” intervention experienced mentoring, networking, and coursework as provided by their institution or usual care [11]. In other words, participants in the “low touch” intervention arm had to seek these opportunities on their own as they were not provided in this intervention arm.

A single Institutional Review Board at the University of Pittsburgh approved the protocol. Participants provided informed consent electronically. Recruitment for Building Up first occurred at the institutional level in which institutions were approached to be a part of the trial [11]. After institutions agreed to participate in Building Up, each institution was responsible for recruiting underrepresented post-doctoral fellows and early-career faculty members at their own institution [11]. The study statistician used block randomization to randomize institutions to receive either the high- or low-touch intervention. Institutions were included in the Building Up study if they successfully recruited between 3 and 12 participants.

### Demographic measures

Participants were asked to report their gender, race, ethnicity, highest degree achieved, and career stage. Race and ethnicity category response options are described in Supplemental Table 1 [12]. “Other” highest degree achieved included MD/PhD, PharmD, PsyD, DDS/DMD, DVM, or other. Participants were asked to identify their primary mentor and the mentor’s title prior to the start of the trial.

### Science identity

Science identity is the extent to which one views themselves as a “scientist” and therefore acts as such [13]. Science identity was assessed using a validated 5-item questionnaire measuring how much participants think being a scientist is part of their personal identity [14]. Questions included: “I have a strong sense of belonging to the community of scientists,” “I derive great personal satisfaction from working on a team that is doing important research,” “I have come to think of myself as a ’scientist’,” “I feel like I belong in the field of science,” and “The daily work of a scientist is appealing to me [14].” Participants rated each item using a 5-point Likert scale ranging from 1 (“strongly disagree”) to 5 (“strongly agree”). Responses were summed and averaged for a total science identity score, with higher scores indicating higher science identity.

### Mentoring competency assessment

Participants were asked to rate the competency of their mentor in six domains: maintenance of effective communication, alignment of expectations, assessment of understanding, ability to foster independence, ability to address diversity, and promotion of professional development [15]. Participants rated each prompt using a 7-point Likert scale ranging from 1 (“not at all”) to 7 (“extremely skilled”). Scores were averaged for a total competency score in each domain [15]. The six domains are described in detail in Supplemental Table 2.

### Self-efficacy in career advancement

Participants completed the C-Change Faculty Survey Dimensions of the Culture scale, to assess self-efficacy in career advancement [16]. This scale includes three measures assessing the belief that advancement is open to them, confidence in career progression, and confidence in overcoming professional barriers.

### Career commitment

Career commitment was measured via two components: intent to continue training to conduct research and intent to continue to study biomedical research [17]. Participants were asked to rate their likelihood of continuing research training and likelihood of continuing to study in a field related to biomedical sciences. Participants rated each item using a 5-point Likert scale ranging from 1 (“definitely will not”) to 5 (“definitely will”). Due to the small number of participants in each group, we collapsed response options for each question into two categories. Individuals who answered “definitely will” and “likely will” were defined as having career commitment (i.e., yes). Individuals who answered “will or will not,” “likely will not,” and “definitely will not” were defined as not having career commitment (i.e., no).

### Statistical analysis

We used SAS version 9.4 (SAS Institute, Cary, NC, USA) for all analyses. Reported p-values are two-tailed; p-values<0.05 were deemed statistically significant. We did not control for multiple comparisons as this was an exploratory analysis [18].

Participant characteristics are reported as medians and 25^th^ and 75^th^ percentiles for continuous data and frequencies and percentages for categorical data.

Separate unadjusted multinomial logistic regression models were conducted to determine associations of each demographic or other characteristic (i.e., science identity and mentoring competency) with each measure of self-efficacy in career advancement. Separate unadjusted logistic regression models were conducted to determine associations of each demographic or other characteristic with each measure of career commitment.

Adjusted multinomial logistic regression was used to identify demographic and other characteristics that were independently associated with feeling as if advancement was open to them. Adjusted multinomial logistic regression was then repeated with confidence in career progression and confidence in overcoming professional barriers as outcome variables in separate models. Adjusted logistic regression was used to identify demographic and other characteristics that were independently associated with both career commitment measures. Variables that were included in each model are summarized in Supplemental Table 3. Variables were entered into single multivariable models with adjustment for gender and race/ethnicity (which were forced into the models because race and gender identity are associated with retention in the biomedical sciences [19]) and retained via backward stepwise elimination if *p* < 0.10. Due to small sample sizes across response strata, career commitment measures were not included as independent variables in the unadjusted or adjusted multinomial logistic regression models where confidence in career progression or confidence in overcoming professional barriers were the dependent variable [20].

## Results

### Cohort characteristics

**Figure 1.**
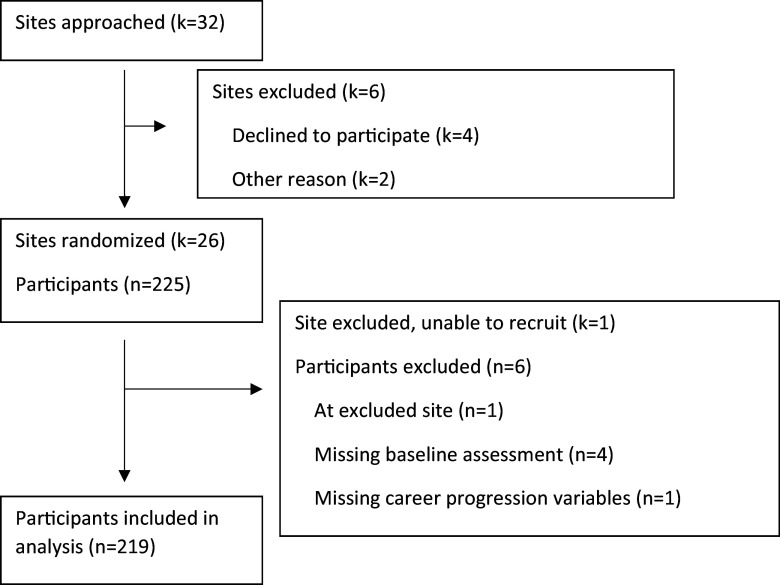
Institution and participant flow diagram for the Building Up a Diverse Biomedical Research Workforce trial.

Two hundred and nineteen individuals (98%) completed the pre-intervention survey and were included in the analyses (Fig. 1). Characteristics of the cohort are summarized in Table 1. Eighty percent of the cohort identified as female, 34% identified as Hispanic/Latinx, 33% identified as non-Hispanic/Latinx Black, 59% had a PhD, and 53% were early-career faculty. No Building Up participants endorsed American Indian, Alaska Native, Native Hawaiian, or Other Pacific Islander as the only racial category that best described them. Fifteen participants identified as multiracial and two as Middle Eastern or North African. The median science identity score was 4.0. The median mentoring competency score was 4.8. Nearly 13% of individuals strongly agreed that advancement was open to them. Nineteen percent of participants strongly agreed that they were confident in their career progression and 16% strongly agreed that they were confident in overcoming professional barriers. Fifty-five percent of individuals answered that they definitely will continue research training and 63% answered that they definitely will continue studying in a field related to the biomedical sciences. Sixty-nine percent of participant mentors were professors, 23% were associate professors, 7% were assistant professors, and 1% did not have an academic appointment.

**Table 1.** Characteristics of underrepresented post-doctoral fellows and early-career faculty, Building Up a Diverse Biomedical Research Workforce trial

Characteristic (n = 219)	No. (%)^a^
Age (median, 25^th^–75^th^ percentile)	36 (33–40)
Gender	
Identifies as male	43 (19.6)
Identifies as female	176 (80.4)
Race/ethnicity	
Hispanic/Latinx	75 (34.3)
Non-Hispanic/Latinx	
White	28 (12.8)
Black	73 (33.3)
Asian	26 (11.9)
Middle Eastern or North African and Multi–Racial	17 (7.8)
Type of highest degree achieved	
MD	68 (31.1)
PhD	129 (58.9)
Other	22 (10.1)
Career stage	
Post-doctoral fellow	102 (46.8)
Faculty	116 (53.2)
Science Identity (median, 25^th^–75^th^ percentile)	4.0 (3.4–4.6)
Range	1.0–5.0
Mentoring Competency Score (median, 25^th^–75^th^ percentile)	4.8 (3.7–5.8)
Range	1.0–7.0
Mentoring that (median, 25^th^–75^th^ percentile)	
Maintains effective communication	5.5 (4.5–6.3)
Aligns expectations	5.2 (4.2–6.0)
Assesses understanding	5.7 (4.3–6.0)
Fosters independence	5.4 (4.2–6.2)
Addresses diversity	5.0 (4.0–6.0)
Promotes professional development	5.2 (4.0–6.0)
*Self-efficacy in career advancement*	
Advancement is open to me	
Strongly agree	28 (12.8)
Agree	74 (33.8)
Neither agree nor disagree	50 (22.8)
Disagree	51 (23.3)
Strongly disagree	16 (7.3)
Confident in career progression	
Strongly agree	41 (18.7)
Agree	101 (46.1)
Neither agree nor disagree	52 (23.7)
Disagree	22 (10.1)
Strongly disagree	2 (1.4)
Confident in overcoming professional barriers	
Strongly agree	34 (15.5)
Agree	106 (48.4)
Neither agree nor disagree	54 (24.7)
Disagree	21 (9.6)
Strongly disagree	4 (1.8)
*Career commitment*	
Intent to continue research training	
Definitely will	128 (54.5)
Likely will	64 (29.2)
Will or will not	20 (9.1)
Likely will not	2 (3.2)
Definitely will not	0 (0.0)
Intent to continue studying in a field related to biomedical sciences	
Definitely will	137 (63.1)
Likely will	47 (21.7)
Will or will not	18 (8.3)
Likely will not	9 (4.2)
Definitely will not	6 (2.8)

a Unless otherwise specified. The number of participants across categories may not sum to the total due to missing data.

### Self-efficacy in career advancement

Unadjusted associations between characteristics of the cohort and self-efficacy in career advancement outcomes are summarized in Supplemental Tables 4-5.

In adjusted models, those with a mentor that addressed diversity had higher odds of [OR: 1.69, 95% CI: (1.34, 2.13); *p* < .001] believing that advancement was open to them (Table 2). Having a higher science identity score [OR: 4.02 per 1 point higher, 95% CI: (1.73, 9.31); *p* = 0.001] and a mentor that fostered independence [OR: 1.78, 95% CI: (1.20, 2.63); *p* = 0.02] were independently associated with confidence in career progression (Table 3). A higher science identity score [OR: 2.32 per 1 point higher, 95% CI: (1.00, 5.36); *p* = 0.01] was independently associated with stronger confidence in overcoming professional barriers (Table 3).

**Table 2.** Adjusted associations between characteristics of underrepresented post-doctoral fellows and early-career faculty and belief that advancement is open to them

	Strongly agree/Agree	Neither agree nor disagree	
	AOR^b^ (95% CI)	AOR^b^ (95% CI)	*P*
Gender			0.25
Identifies as male	2.02 (0.77, 5.26)	1.10 (0.34, 3.53)	
Identifies as female	1.0 (ref.)	1.0 (ref.)	
Race/ethnicity			0.33
Hispanic/Latinx	0.74 (0.28, 1.99)	0.66 (0.19, 2.27)	
Non-Hispanic/Latinx White or Asian	1.0 (ref.)	1.0 (ref.)	
Non-Hispanic/Latinx Black	0.78 (0.28, 2.17)	1.97 (0.61, 6.33)	
Middle Eastern or North African and Multi-Racial	0.98 (0.14, 6.88)	1.69 (0.19, 14.8)	
Mentoring that, per 1 point higher			
Addresses diversity	1.69 (1.34, 2.13)	1.62 (1.23, 2.13)	< 0.001

AOR = adjusted odds ratio.

a Response options are listed in Supplemental Table 2.

b Gender and race/ethnicity forced in the model.

**Table 3.** Adjusted associations between characteristics of underrepresented post-doctoral fellows and early-career faculty, confidence in career progression, and confidence in overcoming professional barriers

	Confident in career progression (Ref=Strongly disagree/Disagree)^a^				Confident in overcoming professional barriers (Ref=Strongly disagree/Disagree)^a^			
	Strongly agree	Agree	Neither agree nor disagree		Strongly agree	Agree	Neither agree nor disagree	
	AOR^b^ (95% CI)	AOR^b^ (95% CI)	AOR^b^ (95% CI)	*P*	AOR^b^ (95% CI)	AOR^b^ (95% CI)	AOR^b^ (95% CI)	*P*
Gender				0.77				0.76
Identifies as male	1.29 (0.33, 5.08)	0.79 (0.23, 2.64)	0.90 (0.24, 3.36)		0.60 (0.15, 2.36)	0.56 (0.19, 1.67)	0.70 (0.20, 2.44)	
Identifies as female	1.0 (ref.)	1.0 (ref.)	1.0 (ref.)		1.0 (ref.)	1.0 (ref.)	1.0 (ref.)	
Race/ethnicity				0.62				0.52
Hispanic/Latinx	0.45 (0.11, 1.97)	0.78 (0.22, 2.74)	0.48 (0.12, 1.90)		0.36 (0.08, 1.75)	0.72 (0.19, 2.69)	0.48 (0.11, 2.08)	
Non-Hispanic/Latinx White or Asian	1.0 (ref.)	1.0 (ref.)	1.0 (ref.)		1.0 (ref.)	1.0 (ref.)	1.0 (ref.)	
Non-Hispanic/Latinx Black	1.15 (0.24, 5.58)	1.49 (0.38, 5.90)	1.27 (0.30, 5.43)		1.03 (0.20, 5.38)	1.51 (0.36, 6.34)	1.14 (0.24, 5.42)	
Middle Eastern or North African and Multi-Racial	1.65 (0.12, 23.7)	1.39 (0.12, 16.1)	3.19 (0.28, 35.9)		3.58 (0.23, 56.6)	0.69 (0.05, 10.5)	1.13 (0.07, 19.2)	
Science identity, per 1 point higher	4.02 (1.73, 9.31)	1.48 (0.80, 2.74)	0.95 (0.50, 1.83)	0.001	2.32 (1.00, 5.36)	1.12 (0.61, 2.06)	0.68 (0.34, 1.34)	0.01
Mentoring that, per 1 point higher								
Fosters independence	1.78 (1.20, 2.63)	1.56 (1.15, 2.12)	1.46 (1.04, 2.04)	0.02	–	–	–	
Addresses diversity	–	–	–		1.48 (1.04, 2.09)	1.21 (0.93, 1.59)	1.48 (1.07, 2.03)	0.06

AOR = adjusted odds ratio.

a Response options are listed in Supplemental Table 2.

b Gender and race/ethnicity forced in the model.

### Career commitment

Unadjusted associations between characteristics of the cohort and career commitment outcomes are summarized in Supplemental Table 6.

Higher age [OR: 2.29 per every 5-year increase, 95% CI: (1.22, 4.31); *p* = 0.01] and having a higher science identity score [OR: 4.20 per 1 point higher, 95% CI: (1.95, 9.04); *p* < .001] were independently associated with intent to continue research training. Having a mentor that maintained effective communication [OR: 0.37, 95% CI: (0.15, 0.92); *p* = 0.03] and assessed understanding [OR: 0.48, 95% CI: (0.24, 0.95); *p* = 0.04] were independently associated with a lower likelihood of continuing research training (Table 4). Higher science identity score [OR: 3.28 per 1 point higher, 95% CI: (1.80, 5.96); *p* < .001] was independently associated with intent to continue studying in a field related to the biomedical sciences (Table 4).

**Table 4.** Adjusted associations between characteristics of underrepresented post-doctoral fellows and early-career faculty and career commitment

	Intent to continue research training (Ref=No)^a^		Intent to continue studying in a field related to biomedical sciences (Ref=No)^a^	
	AOR^b^ (95% CI)	*P*	AOR^b^ (95% CI)	*P*
Age, per every 5 years higher	2.29 (1.22, 4.31)	0.01	1.58 (0.97, 2.56)	0.06
Gender		0.43		0.17
Identifies as male	0.59 (0.16, 2.19)		0.46 (0.15, 1.39)	
Identifies as female	1.0 (ref.)		1.0 (ref.)	
Race/ethnicity		0.46		0.71
Hispanic/Latinx	0.23 (0.04, 1.47)		0.99 (0.23, 4.23)	
Non-Hispanic/Latinx White or Asian	1.0 (ref.)		1.0 (ref.)	
Non-Hispanic/Latinx Black	0.45 (0.07, 2.84)		0.88 (0.23, 3.31)	
Middle Eastern or North African and Multi-Racial	0.31 (0.03, 3.30)		0.42 (0.08, 2.26)	
Science identity, per 1 point higher	4.20 (1.95, 9.04)	< 0.001	3.28 (1.80, 5.96)	< 0.001
Mentoring that, per 1 point higher				
Maintains effective communication	0.37 (0.15, 0.92)	0.03		
Aligns expectations	2.19 (0.98, 4.87)	0.06	–	
Assesses understanding	0.48 (0.24, 0.95)	0.04	–	
Fosters independence	2.29 (0.97, 5.40)	0.06		

AOR = adjusted odds ratio.

a Response options are listed in Supplemental Table 2.

b Gender and race/ethnicity forced in the model.

## Discussion

We found that stronger science identity was significantly associated with self-efficacy in career advancement and career commitment among post-doctoral fellows and early-career faculty from underrepresented groups. We also found that mentorship that addressed diversity and fostered independence was significantly associated with self-efficacy in career advancement among post-doctoral fellows and early-career faculty from underrepresented groups. These are consistent with previous findings that show that mentor mindset (e.g., addressing diversity, understanding, and facilitating identity work) has a significant effect on the self-efficacy and work engagement of mentees [21].

Our findings indicate that mentoring that addresses diversity is associated with self-efficacy in career advancement in this cohort. Mentoring that addresses diversity may inspire and build confidence among underrepresented mentees, prioritize exposing underrepresented mentees to individuals from underrepresented groups in leadership positions, and allow for important identity-related work to take place within the mentoring relationship. Findings in undergraduate programs show that mentors taught to address diversity are more sensitive in how they approach race/ethnicity-related topics and more likely to create safe spaces for mentees to speak about these topics [22]. Prior research shows that diverse mentoring teams for faculty from groups underrepresented in medicine improve career progression and ability to overcome obstacles in career advancement [3]. Our findings also support research that shows that underrepresented faculty members and post-doctoral fellows believe that universal access to diverse mentorship would expedite their career progression and ability to advance at their institution [23]. Unfortunately, we did not collect information on the specific ways in which mentors addressed diversity. Future research should identify specific aspects of addressing diversity in mentoring relationships that are associated with self-efficacy in career advancement among underrepresented post-doctoral fellows and early-career faculty.

Science identity was associated with self-efficacy in career advancement and career commitment. Previous literature shows that identity development takes place via “transformative learning”—a process in which individuals must shed parts of their original identity to redefine or grow their identity [24]. A stronger sense of science identity can only be achieved through the process of transformative learning [24]. What triggers transformative learning and identity development in researchers from underrepresented backgrounds is still not well understood. Previous literature suggests that peer mentorship plays a significant role in identity development, including science identity, in mentees from underrepresented groups [25]. The role that formal mentoring teams play in identity development is still unclear, although some research suggests a relationship between mentoring as identity work and positive career outcomes [6]. Future research should investigate the impact of mentorship on science identity development among early-career researchers from underrepresented groups. In particular, stronger science identity in mentors may be associated with stronger science identity among underrepresented mentees. Understanding these relationships better will help future development of interventions to increase self-efficacy in career advancement among and retention of underrepresented post-doctoral fellows and early-career faculty in the biomedical research workforce.

Nearly all participants in this study were committed to continuing research training. This is not surprising considering our previous research showing that underrepresented post-doctoral fellows and early-career faculty have high levels of grit [26]. Grit, which consists of perseverance and consistency of interest, has been shown to positively impact career success and goal achievement [27]. The more grit an individual has, the more likely they are to pursue career goals and achieve career success [27]. Our cohort is “very gritty [26],” which may explain why no one in this cohort indicated that they definitely will not continue training to conduct research. Although this cohort has a high level of grit [26], individuals from underrepresented backgrounds face systemic discrimination, lack of representation in the biomedical workforce, and stereotypes [28,29]. Although these obstacles can negatively impact career commitment, in our cohort, a small percentage of individuals “strongly agreed” that they were confident in their ability to progress in their career (19%) or overcome professional barriers (16%), and most participants were committed to continuing research training and studying in a field related to biomedical science.

Our data were collected during the COVID-19 pandemic and Racial Justice Movement; therefore, our results are difficult to compare to previous findings. The psychological distress that underrepresented post-doctoral and early-career faculty faced during this time was likely escalated and may have negatively impacted their self-efficacy in career advancement and career commitment, especially because a sizable minority of underrepresented post-doctoral fellows and early-career faculty reported lower research productivity [30]. Furthermore, since this was a cross-sectional analysis, we could not assess causal associations. The cohort was also majority female, which limits the generalizability of our findings because our sample is not representative of underrepresented researchers across the nation. Additionally, gender differences in levels of science identity and self-efficacy in career advancement may have impacted our results [31]. Our study explores the effects of individual characteristics on career progression among underrepresented researchers without taking into account institutional-level characteristics that likely impact career progression among underrepresented post-doctoral fellows and early-career faculty. The role of institutional climate and inclusivity on self-efficacy of career advancement and career commitment should be further explored. Lastly, we collected very limited data about participants’ mentors. Our results show that mentor identity is important to consider when investigating mentees from underrepresented groups.

Our study adds to current literature that assesses factors associated with self-efficacy in career advancement and career commitment among post-doctoral fellows and early-career researchers from groups underrepresented in biomedical sciences. The cohort includes a large number of underrepresented post-doctoral fellows and early-career faculty from 25 different academic institutions across the United States participating in the Building Up trial. Because institutions support diversity at different levels, it is possible that self-efficacy of career advancement and career commitment varied by institution. We did not analyze self-efficacy of career advancement or career commitment by institution as this was not a pre-specified aim of this study and we were underpowered to do so.

## Conclusions

In this study, we found that mentorship and science identity are significantly associated with self-efficacy in career advancement and career commitment among post-doctoral fellows and early-career faculty from underrepresented groups. These data can be used to develop effective interventions to retain and support the career progression of researchers underrepresented in the biomedical sciences.

## Supporting information

Thakar et al. supplementary material Thakar et al. supplementary material

## Supplementary material

The supplementary material for this article can be found at https://doi.org/10.1017/cts.2024.504.
