# Supplementary material for: The impact of inclusive mentoring and identity work on self-efficacy in career advancement and career commitment among underrepresented early-career faculty and post-doctoral fellows
Source: J Clin Transl Sci. 2024 Mar 22;8(1):e61. doi: 10.1017/cts.2024.504 (PMC11036437; doi:10.1017/cts.2024.504)
Supplement: Thakar et al. supplementary material [file S2059866124005041sup001.pdf]

Supplemental Figure 1. Geographic location of all 25 academic institutions involved in Building Up a Diverse Biomedical Research Workforce Trial

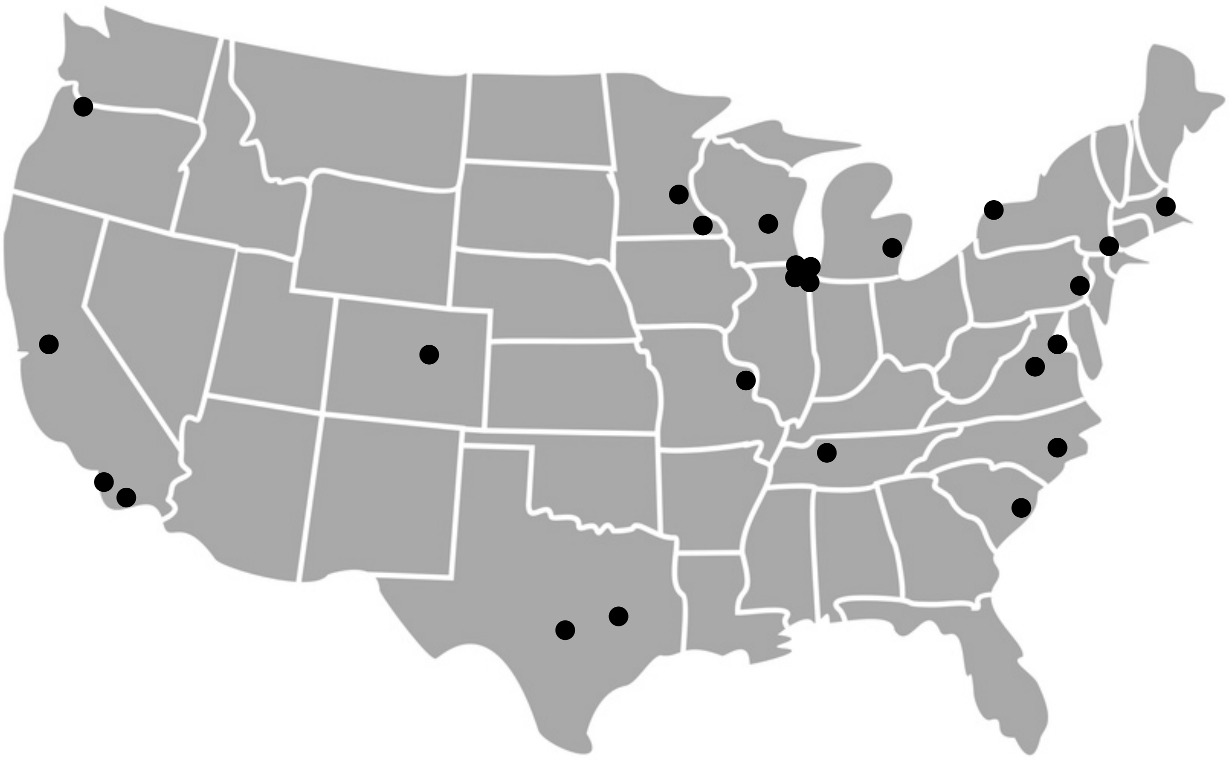

Albert Einstein College of Medicine/Montefiore Medical Center
Children's National Medical Center/The George Washington University
Loyola University Medical Center
Massachusetts General Hospital
Mayo Clinic Rochester
Medical University of South Carolina
Northwestern University
Oregon Health and Science University
Rush University Medical Center
Texas A&M University
Tufts University Health Sciences
University of Buffalo
University of California, Davis
University of Chicago
University of Colorado Denver Anschutz Medical Campus
University of Michigan
University of Minnesota
University of Pennsylvania Perelman School of Medicine
University of Southern California
University of Texas Health Science Center at San Antonio/University of Texas at Austin
University of Virginia
University of Wisconsin, Madison
Vanderbilt University Medical Center
Washington University at St. Louis School of Medicine

Supplemental Table 1. Race and ethnicity question[a] used in Building Up a Diverse Biomedical Research Workforce Trial

Which categories best describe you? Select all boxes that apply. Note, you may report more than one group.
1. White – For example, German, Irish, English, Italian, Polish, French, etc.<br>2. Hispanic, Latinx, or Spanish origin – For example, Mexican or Mexican American, Puerto Rican, Cuban, Salvadoran, Dominican, Columbian, etc.<br>3. Black or African American – For example, African American, Jamaican, Haitian, Nigerian, Ethiopian, Somali, etc.<br>4. Asian – For example, Chinese, Filipino, Asian Indian, Vietnamese, Korean, Japanese, Pakistani, etc.<br>5. American Indian or Alaska Native – For example, Navajo Nation, Blackfeet Tribe, Mayan, Aztec, Native Village of Barrow Inuplat Traditional Government, Nome Eskimo Community, etc.<br>6. Middle Eastern or North African – For example, Lebanese, Iranian, Egyptian, Syrian, Moroccan, Algerian, etc.<br>7. Native Hawaiian or Other Pacific Islander – For example, Native Hawaiian, Samoan, Chamorro, Tongan, Fijian, Marshallese, etc.<br>8. Some other race, ethnicity, or origin

[a] Maccalla NMG, Gutierrez, A., Zhong, S., Wallace, S.P., & McCreath H.E. *TECHNICAL REPORT: Evaluation of Post-secondary Student Outcomes: Underrepresented (URG) and Well-Represented (WRG) Group Variable Construction in the Enhance Diversity Study using the November 2019 NIH Guidelines*. 2020.

Supplemental Table 2. Mentoring Competency Assessment domains

Maintains effective communication
Active listening
Providing you constructive feedback
Establishing a relationship based on trust with you
Identifying and accommodating different communication styles
Employing strategies to improve communication with you
Aligns expectations
Working with you to set clear expectations of the mentoring relationship
Aligning his/her expectations with your own
Considering how personal and professional differences may impact expectations
Working with you to set research goals
Helping you develop strategies to meet research goals
Assesses understanding
Accurately estimating your level of scientific knowledge
Accurately estimating your ability to conduct research
Employing strategies to enhance your understanding of the research
Fosters independence
Motivating you
Building your confidence
Stimulating your creativity
Acknowledging your professional contributions
Negotiating a path to professional independence with you
Addresses diversity
Taking into account the biases and prejudices s/he brings to your mentor/mentee relationship
Working effectively with mentees whose personal background is different from his/her own (age, race, gender, class, region, culture, religion, family composition, etc.)
Promotes professional development
Helping you network effectively
Helping you set career goals
Helping you balance work with your personal life
Understanding his/her impact as a role model for you
Helping you acquire resources (e.g. grants, etc)

Supplemental Table 3. Independent and dependent variables tested and retained in each adjusted model

	Self-efficacy in career advancement			*Career commitment*	
	Model 1[a]	Model 2[a]	Model 3[a]	Model 4[b]	Model 5[b]
**Dependent variable**	Advancement is open to me	Confident in career progression	Confident in overcoming professional barriers	Intent to continue research training	Intent to continue studying in a field related to biomedical sciences
**Independent variables**	Age<br>Race/ethnicity[c]<br>Gender[c]<br>Type of highest degree achieved<br>Career stage<br>Science identity score<br>Mentoring that<br>Maintains effective communication<br>Aligns expectations<br>Assesses understanding<br>Fosters independence<br>Addresses diversity*<br>Promotes professional development<br>Intent to continue research training<br>Intent to continue studying in a field related to biomedical sciences	Age<br>Race/ethnicity[c]<br>Gender[c]<br>Type of highest degree achieved<br>Career stage<br>Science identity score*<br>Mentoring that<br>Maintains effective communication<br>Aligns expectations<br>Assesses understanding<br>Fosters independence*<br>Addresses diversity<br>Promotes professional development	Age<br>Race/ethnicity[c]<br>Gender[c]<br>Type of highest degree achieved<br>Career stage<br>Science identity score*<br>Mentoring that<br>Maintains effective communication<br>Aligns expectations<br>Assesses understanding<br>Fosters independence<br>Addresses diversity*<br>Promotes professional development	Age<br>Race/ethnicity[c]<br>Gender[c]<br>Type of highest degree achieved<br>Career stage*<br>Science identity score*<br>Mentoring that<br>Maintains effective communication*<br>Aligns expectations*<br>Assesses understanding*<br>Fosters independence<br>Addresses diversity<br>Promotes professional development*	Age<br>Race/ethnicity[c]<br>Gender[c]<br>Type of highest degree achieved<br>Career stage<br>Science identity score*<br>Mentoring that<br>Maintains effective communication<br>Aligns expectations<br>Assesses understanding<br>Fosters independence<br>Addresses diversity<br>Promotes professional development

*Variable was retained in the model due to $p<0.10$

[a]Multinomial logistic regression, [b]Logistic regression, [c]Gender identity and race/ethnicity were retained in all models regardless of p-value

Career commitment variables were not included as independent variables in which confidence in career progression or confidence in overcoming professional barriers were outcomes because of small sample sizes across strata.

Supplemental Table 4. Unadjusted associations between characteristics of underrepresented post-doctoral fellows and early-career faculty and belief that advancement is open to them

	Advancement is open to me (Ref=Strongly disagree/Disagree)[a]		
	Strongly agree/Agree	Neither agree nor disagree	
Characteristic	OR (95% CI)	OR (95% CI)	*P*
Age, per 5 years higher	1.10 (0.83, 1.45)	1.13 (0.80, 1.57)	0.75
Gender			0.12
Identifies as male	1.98 (0.88, 4.43)	0.93 (0.33, 2.64)	
Identifies as female	1.0 (ref.)	1.0 (ref.)	
Race/ethnicity			0.27
Hispanic/Latinx	0.84 (0.37, 1.90)	0.66 (0.23, 1.91)	
Non-Hispanic/Latinx White or Asian	1.0 (ref.)	1.0 (ref.)	
Non-Hispanic/Latinx Black	0.50 (0.22, 1.17)	1.12 (0.42, 2.97)	
Middle Eastern or North African and Multi-Racial	0.68 (0.18, 2.51)	1.27 (0.29, 5.53)	
Type of highest degree achieved			0.25
MD	0.77 (0.23, 2.54)	1.56 (0.36, 6.80)	
PhD	0.48 (0.16, 1.44)	0.71 (0.17, 2.87)	
Other	1.0 (ref.)	1.0 (ref.)	
Career stage			0.60
Post-doctoral fellow	1.0 (ref.)	1.0 (ref.)	
Faculty	1.32 (0.71, 2.45)	1.00 (0.48, 2.09)	
Science identity, per 1 point higher	1.28 (0.87, 1.88)	1.39 (0.87, 2.22)	0.30
Mentoring that, per 1 point higher			
Maintains effective communication	1.70 (1.29, 2.24)	1.53 (1.12, 2.10)	<.001
Aligns expectations	1.67 (1.29, 2.16)	1.57 (1.17, 2.11)	<.001
Assesses understanding	1.63 (1.29, 2.08)	1.44 (1.11, 1.87)	<.001
Fosters independence	1.61 (1.27, 2.04)	1.49 (1.13, 1.97)	<.001
Addresses diversity	1.72 (1.36, 2.16)	1.55 (1.19, 2.02)	<.001
Promotes professional development	1.62 (1.27, 2.07)	1.38 (1.05, 1.82)	<.001
Intent to continue research training			0.12
Yes	2.49 (1.00, 6.20)	2.17 (0.72, 6.54)	
No	1.0 (ref.)	1.0 (ref.)	
Intent to continue studying in a field related to biomedical sciences			0.22
Yes	2.12 (0.86, 5.25)	2.02 (0.66, 6.20)	
No	1.0 (ref.)	1.0 (ref.)	

OR, odds ratio

Supplemental Table 5. Unadjusted associations between characteristics of underrepresented post-doctoral fellows and early-career faculty, confidence in career progression, and confidence in overcoming professional barriers

	Confident in career progression (Ref=Strongly disagree/Disagree)[a]				Confident in overcoming professional barriers (Ref=Strongly disagree/Disagree)[a]			
	Strongly agree	Agree	Neither agree nor disagree		Strongly agree	Agree	Neither agree nor disagree	
Characteristics	OR (95% CI)	OR (95% CI)	OR (95% CI)	*P*	OR (95% CI)	OR (95% CI)	OR (95% CI)	*P*
Age, per 5 years higher	1.04 (0.67, 1.63)	1.02 (0.69, 1.51)	0.75 (0.48, 1.17)	0.28	0.95 (0.61, 1.50)	0.91 (0.62, 1.33)	0.81 (0.54, 1.24)	0.77
Gender				0.53				0.46
Identifies as male	1.16 (0.37, 3.66)	0.65 (0.23, 1.86)	0.66 (0.21, 2.13)		0.46 (0.14, 1.54)	0.47 (0.18, 1.25)	0.48 (0.16, 1.43)	
Identifies as female	1.0 (ref.)	1.0 (ref.)	1.0 (ref.)		1.0 (ref.)	1.0 (ref.)	1.0 (ref.)	
Race/ethnicity				0.45				0.86
Hispanic/Latinx	0.49 (0.13, 1.84)	0.77 (0.24, 2.50)	0.30 (0.11, 1.44)		0.51 (0.13, 2.00)	0.60 (0.19, 1.92)	0.44 (0.13, 1.57)	
Non-Hispanic/Latinx White or Asian	1.0 (ref.)	1.0 (ref.)	1.0 (ref.)		1.0 (ref.)	1.0 (ref.)	1.0 (ref.)	
Non-Hispanic/Latinx Black	0.68 (0.17, 2.65)	0.92 (0.27, 3.18)	0.80 (0.22, 3.00)		1.02 (0.23, 4.47)	1.30 (0.36, 4.72)	0.94 (0.24, 3.74)	
Middle Eastern or North African and Multi-Racial	1.25 (0.10, 15.1)	1.09 (0.10, 11.5)	2.86 (0.28, 29.0)		0.83 (0.10, 6.78)	0.60 (0.09, 3.88)	1.00 (0.15, 6.64)	
Type of highest degree achieved				0.64				0.85
MD	0.33 (0.03, 3.58)	0.46 (0.05, 4.21)	0.79 (0.07, 8.52)		2.00 (0.29, 13.7)	1.98 (0.40, 9.77)	2.00 (0.37, 10.9)	
PhD	0.29 (0.03, 2.69)	0.26 (0.03, 2.13)	0.40 (0.04, 3.90)		1.24 (0.22, 6.92)	1.11 (0.28, 4.50)	0.82 (0.18, 3.73)	
Other	1.0 (ref.)	1.0 (ref.)	1.0 (ref.)		1.0 (ref.)	1.0 (ref.)	1.0 (ref.)	
Career stage				0.30				0.61
Post-doctoral fellow	1.0 (ref.)	1.0 (ref.)	1.0 (ref.)		1.0 (ref.)	1.0 (ref.)	1.0 (ref.)	
Faculty	0.74 (0.27, 2.03)	0.90 (0.37, 2.19)	0.49 (0.19, 1.29)		0.73 (0.26, 2.05)	1.23 (0.51, 2.95)	0.99 (0.39, 2.57)	
Science identity, per 1 point higher	3.19 (1.56, 6.55)	1.38 (0.80, 2.36)	0.94 (0.53, 1.66)	0.002	1.78 (0.86, 3.68)	1.04 (0.59, 1.83)	0.66 (0.36, 1.20)	0.01
Mentoring that, per 1 point higher								
Maintains effective communication	1.67 (1.11, 2.50)	1.52 (1.09, 2.11)	1.28 (0.91, 1.81)	0.04	1.67 (1.09, 2.55)	1.37 (1.00, 1.89)	1.40 (0.98, 2.00)	0.10
Aligns expectations	1.67 (1.14, 2.45)	1.45 (1.07, 1.96)	1.23 (0.89, 1.70)	0.03	1.79 (1.18, 2.70)	1.38 (1.03, 1.86)	1.39 (1.00, 1.93)	0.04
Assesses understanding	1.55 (1.10, 2.19)	1.39 (1.05, 1.83)	1.25 (0.93, 1.69)	0.06	1.39 (0.97, 2.00)	1.17 (0.89, 1.54)	1.19 (0.88, 1.60)	0.35
Fosters independence	1.94 (1.33, 2.84)	1.58 (1.17, 2.14)	1.43 (1.03, 1.96)	0.004	1.68 (1.13, 2.48)	1.24 (0.93, 1.65)	1.29 (0.94, 1.77)	0.08
Addresses diversity	1.44 (1.05, 1.97)	1.27 (0.99, 1.64)	1.31 (0.98, 1.76)	0.13	1.44 (1.03, 2.01)	1.18 (0.92, 1.53)	1.37 (1.01, 1.85)	0.10
Promotes professional development	1.77 (1.22, 2.58)	1.62 (1.19, 2.21)	1.39 (1.01, 1.94)	0.01	1.44 (0.99, 2.12)	1.20 (0.89, 1.62)	1.13 (0.82, 1.56)	0.29

OR, odds ratio

Supplemental Table 6. Unadjusted associations between characteristics of underrepresented post-doctoral fellows and early-career faculty and career commitment

	Intent to continue research training (Ref=No)[a]		Intent to continue studying in a field related to biomedical sciences (Ref=No)[a]	
Characteristic	OR (95% CI)	*P*	OR (95% CI)	*P*
Age, per 5 years higher	1.71 (1.10, 2.67)	0.02	1.59 (1.03, 2.45)	0.04
Gender		0.33		0.73
Identifies as male	0.63 (0.25, 1.60)		0.84 (0.32, 2.24)	
Identifies as female	1.0 (ref.)		1.0 (ref.)	
Race/ethnicity		0.35		0.23
Hispanic/Latinx	0.38 (0.10, 1.46)		0.79 (0.22, 2.86)	
Non-Hispanic/Latinx White or Asian	1.0 (ref.)		1.0 (ref.)	
Non-Hispanic/Latinx Black	0.30 (0.08, 1.12)		0.42 (0.13, 1.38)	
Middle Eastern or North African and Multi-Racial	0.44 (0.07, 2.89)		0.28 (0.06, 1.26)	
Type of highest degree achieved		0.22		0.26
MD	0.22 (0.03, 1.82)		0.22 (0.03, 1.82)	
PhD	0.39 (0.05, 3.14)		0.36 (0.05, 2.92)	
Other	1.0 (ref.)		1.0 (ref.)	
Career stage		0.03		0.07
Post-doctoral fellow	1.0 (ref.)		1.0 (ref.)	
Faculty	2.55 (1.09, 5.96)		2.14 (0.93, 4.93)	
Science identity, per 1 point higher	3.10 (1.85, 5.21)	<.001	3.10 (1.85, 5.21)	<.001
Mentoring that, per 1 point higher				
Maintains effective communication	1.33 (0.99, 1.79)	0.06	1.37 (1.01, 1.86)	0.047
Aligns expectations	1.40 (1.07, 1.84)	0.01	1.31 (0.99, 1.72)	0.06
Assesses understanding	1.13 (0.87, 1.47)	0.35	1.19 (0.92, 1.55)	0.19
Fosters independence	1.38 (1.06, 1.79)	0.02	1.30 (0.99, 1.70)	0.06
Addresses diversity	1.16 (0.91, 1.49)	0.24	1.25 (0.97, 1.61)	0.09
Promotes professional development	1.29 (0.98, 1.71)	0.07	1.22 (0.92, 1.62)	0.18

OR, odds ratio
